# The Poor Man’s Book

**Published:** 1858-04

**Authors:** 


### THE POOR MAN'S BOOK.

A FEW Sundays ago, we heard from our minister the expression, "*The Bible is emphatically the poor man's book;*" and on reflection, it occurred to us, that it was a most truthful sentiment. One of the very last recorded warnings in The Old Testament dispensation is directed to "*Those that oppress the hireling in his wages, the widow and the fatherless.*" What could be more beneficent in its aims for the daily laborer, than the imperative injunction of Moses, "*The wages of him that is hired shall not abide with thee all night, until the morning.*" What multitudes of hearts of the poor, in a great city like this, sink within them, when, applying for their wages, they are told to wait a few days, or even until to-morrow, that it is not "*convenient*" now. Nor is it convenient, reader, for that sick child at home, to wait a day or two for medicine; nor for a hungry family, without credit, to wait until to-morrow, for this day's bread—cold, and sleet, and sun, and storm, never wait. The authoritative expression to laborers, "You must wait a day or two," is equivalent to, in cases not a few, "For a day or two you must abide in hunger and cold." How soothingly to the poor in this world's goods, who make the Bible "*their*" Book, is the assurance that its great Hero "had not a place to lay his head;" that he could be touched with the feelings of their infirmities, having been tempted in all points as they were! And as to the physical well-being of the poor, nothing could be more to the purpose, than the numerous injunctions in the *Scriptures*—injunctions of the most specific character as to cleanliness, as to moderation, as to temperance, as to the restraint of evil passions and propensities, and as to the cultivation of the finer feelings of our nature—purity of body, purity of mind.

Anxiety, and privation, and want, hurry multitudes of poor every year to a premature grave. But thrift and Bible religion go together the world over; and its principles, pure, and unadulterated by human traditions, and human expositions, and commentaries, are the only panacea for the cure of disease, and want, and crime; hence it is not at all out of the way in a "Journal of Health" to draw attention to a Book which so largely inculcates attention to the three great foundations of human health and physical well-being—CLEANLINESS, TEMPERANCE, and INDUSTRY.

These things being so, we regard that as a "sanitary measure" which directs public attention to the teachings of the Bible, which seeks to make the Bible a classical book, by inviting the young to read it, and to study its numberless histories, biographies, incidents, and facts. Above all others, do we welcome the books, and praise the men who make them, which are calculated to interest the mass of readers in Bible narratives. The Rev. T. H. STOCKTON, of Philadelphia, has taken an important step in the right direction, in publishing his Periodical New Testament, and his "Book and Journal." The *Religious Intelligencer*, of St. John's, New Brunswick, has a succession of well-written articles, designed to draw the attention of the young to the study of Bible histories. In the same direction do we notice a book from the far, far West, just published, *The Giant Judge; or*, *The Story of Sampson*, *the Hebrew Hercules*, by that eminent divine, the able scholar, and Christian gentleman, W. A. Scott, D. D., of San Francisco. A million-fold more prolific of practical lessons, and of beautiful allusions, is the life of the son of Manoah, than of the mythological Stable-cleaner.

In listening to the lectures of some of the most eminent medical men of Great Britain, Sir Henry Lawrence, and others, we were forcibly struck with their frequent spontaneous and appropriate acknowledgment of the claims of Christianity upon the young men who were attending their teachings, and the apt allusions made to historical incidents in the Bible. And, if our Legislators and Congressmen were to make the Bible classic ground, were to illustrate their sentiments by its teachings, with the reverence becoming the subject, it would not be long before a higher morality would pervade our halls of legislation; and a manly decorum, a Christian courtesy, would take take place of the cowardly assault, the mean intimidation, and the brutal braggadocio.

